# Incidence and characteristics of chronic thromboembolic pulmonary hypertension in Germany

**DOI:** 10.1007/s00392-018-1215-5

**Published:** 2018-02-15

**Authors:** Thorsten Kramm, Heinrike Wilkens, Jan Fuge, Hans-Joachim Schäfers, Stefan Guth, Christoph B. Wiedenroth, Bettina Weingard, Doerte Huscher, David Pittrow, Serghei Cebotari, Marius M. Hoeper, Eckhard Mayer, Karen M. Olsson

**Affiliations:** 1 Kerckhoff Heart and Lung Center, Bad Nauheim, Germany; 2 Clinic for Internal Medicine V-Pneumology, Allergology and Critical Care Medicine, Saarland University Hospital, Homburg, Germany; 3 Department of Respiratory Medicine and German Center of Lung Research (DZL/BREATH), Hannover Medical School, 30623 Hanover, Germany; 4 Department of Thoracic and Cardiovascular Surgery, Saarland University Hospital, Homburg, Germany; 5 Epidemiology Unit, German Rheumatism Research Centre, Berlin, Germany; 6 Institute for Clinical Pharmacology, Medical Faculty, Technical University, Dresden, Germany; 7 Clinic for Cardiothoracic, Vascular and Transplantation Surgery, Hannover Medical School, Hanover, Germany

**Keywords:** Chronic thromboembolic pulmonary hypertension, Incidence, Epidemiology, Pulmonary endarterectomy, Balloon pulmonary angioplasty

## Abstract

**Background:**

The incidence of chronic thromboembolic pulmonary hypertension (CTEPH) is unknown. Previous studies from the United Kingdom and Spain have reported incidence rates of 1.75 and 0.9 per million, respectively. These figures, however, may underestimate the true incidence of CTEPH.

**Methods:**

We prospectively enrolled patients newly diagnosed with CTEPH within 2016 in Germany. Data were obtained from the three German referral centers and from the German branch of COMPERA, a European pulmonary hypertension registry. The CTEPH incidence was calculated based on German population data, and patient characteristics and treatment patterns were described.

**Results:**

A total of 392 patients were newly diagnosed with CTEPH within 2016 in Germany, yielding an incidence of 5.7 new cases per million adults. The (mean ± standard deviation) age was 63.5 ± 15.0 years; males and females were equally affected; 76.3% of the patients had a history of venous thromboembolism. A total of 197 (50.3%) patients underwent pulmonary endarterectomy. Almost all non-operated patients received targeted drug therapy, and 49 patients (25.1% of the non-operated patients) were treated with balloon pulmonary angioplasty.

**Conclusion:**

The incidence of CTEPH in Germany 2016 was 5.7 per million adults and thus higher than previously reported from other countries. Half of the patients were operated while the remaining patients received medical or interventional therapies.

**Clinical trials registration:**

http://www.clinicaltrials.gov NCT02660463 and NCT01347216.

## Introduction

Chronic thromboembolic pulmonary hypertension (CTEPH) is defined by an elevated mean pulmonary artery pressure at rest caused by persistent obstruction of pulmonary arteries despite therapeutic anticoagulation for at least 3 months following pulmonary embolism [1, 2]. Among the various forms of pulmonary hypertension (PH), CTEPH has some unique features including the availability of a variety of treatment options. The majority of patients with CTEPH can be effectively treated, and often cured, by pulmonary endarterectomy (PEA) [3–6]. For patients who are not candidates for surgery, medical and interventional therapies are available [7–11].

The epidemiology of CTEPH is largely unknown. The annual incidence of acute pulmonary embolism ranges from 750 to 2700 per million adults [12–14]. Several studies on the risk of developing CTEPH in survivors of acute pulmonary embolism came up with figures ranging from 1 to 9% [15–18]. A recent meta-analysis of the available data suggested that the incidence of CTEPH in survivors of acute pulmonary embolism is about 3% [19]. Based on these numbers, the expected incidence of CTEPH would be 22.5 to 81 per million adults. In contrast, the reported numbers of patients with an established diagnosis of CTEPH are substantially smaller. Two nationwide registries have assessed the incidence of CTEPH: In the United Kingdom, the CTEPH incidence was 1.75 per million population [20]; in Spain, it was 0.9 per million adults [21].

In the present study, we prospectively assessed the incidence and characteristics of CTEPH in 2016 in Germany.

## Methods and patients

We designed a prospective registry to obtain data from the three German CTEPH referral centers (Kerckhoff Heart and Lung Center, Bad Nauheim, Hannover Medical School and Saarland University Medical Center, Homburg) and from Comparative, Prospective Registry of Newly Initiated Therapies for Pulmonary Hypertension (COMPERA), a European-based PH registry which includes adult patients with all forms for PH who receive targeted medical therapy. COMPERA is the official German PH registry and has broad participation from German PH centres [22]. We extracted from the COMPERA database all German patients who had been newly diagnosed in 2016 with CTEPH but who had not been referred to one of the three above-mentioned centers, thereby avoiding double counting. Data were collected at the baseline visit and included demographics, haemodynamics, functional class, diagnostic tools and treatment strategies.

For the present analysis, we included adult patients ≥ 18 years of age with a newly established CTEPH diagnosis between 1 January and 31 December 2016. The diagnostic criteria were in accordance with the current European Guidelines on Pulmonary Hypertension [23]. Patients referred from other countries were not eligible. Although the study included exclusively patients who received their first diagnosis of CTEPH within 2016, the database was locked only on 30 June 2017, to allow for a brief follow-up period focussing on treatment decisions and outcomes of patients who underwent surgery.

The study was approved by the institutional review boards of the participating centers and has been registered under the clinical trials.gov identifier NCT02660463. All patients provided written informed consent.

### Statistical analysis

The data were captured on Excel spreadsheets in the participating centers and were merged with data exported from COMPERA. All descriptive analyses were done with SPSS v25 and no formal statistical analyses were performed.

## Results

In 2016, a total of 392 patients were newly diagnosed with CTEPH in Germany. Bad Nauheim contributed *N* = 234 (59.7%) patients, Homburg *N* = 69 (17.6%), Hannover *N* = 57 (14.5%) and COMPERA *N* = 32 (8.2%). Given the 68.6 million adults living in Germany in 2016 (http://www.destatis.de, website accessed 18 October 2017), the calculated CTEPH incidence was 5.7 per million adults.

The patients’ characteristics are shown in Table 1. The mean age was 63.5 years and the female-to-male ratio was equal. The mean age of the COMPERA patients was 71 years and thus about 8 years higher than the age of the patients referred to the CTEPH centers. Most of the patients presented with moderately or severely impaired exercise capacity at the time of diagnosis.

**Table 1:** Patients characteristics at time of diagnosis in 392 patients with newly diagnosed chronic thromboembolic pulmonary hypertension

	*n* = 392
Age (years), *N* = 392	63.5 ± 15.0
Female/male (%), *N* = 392	50.8/49.2
Body mass index (kg/m^2^); *N* = 389	28.1 ± 6.5
6 min walk distance, *N* = 156	326 ± 121
WHO functional class, *N* = 305	I, 1 (0.3%)
	II, 76 (24.9%)
	III, 182 (59.7%)
	IV, 46 (15.1%)
RAP (mmHg), *N* = 306	9 ± 45
PAPm (mmHg), *N* = 374	43 ± 10
PAWP (mmHg), N = 357	11 ± 4
CO (l/min), *N* = 315	4.6 ± 1.4
CI (l/min/m^2^), *N* = 345	2.5 ± 1.9
PVR (dyn s cm^−5^), *N* = 345	652 ± 396
SvO_2_ (%), *N* = 235	64 ± 8

*RA* right atrial pressure, *PAPm* mean pulmonary artery pressure, *PAWP* pulmonary arterial wedge pressure, *CO* cardiac output, *CI* Cardiac Index, *PVR* pulmonary vascular resistance, *SvO*_*2*_ mixed venous oxygen saturation

76.3% of the patients had a history of venous thromboembolism and 150 (38.3%) had at least one predisposing factor, most commonly thrombophilia (8.2%), malignancy (5.6%), antiphospholipid antibodies (4.6%), cardiac pacemakers (2.6%) and splenectomy (1.5%).

The diagnostic assessment included ventilation–perfusion scintigraphy in 93.8% of the patients. Computed tomography angiography and pulmonary angiography were performed in 88% and 76% of the patients, respectively.

Anticoagulants were used in all patients, predominantly direct oral anticoagulants (51%) and vitamin K antagonists (46.2%). A small proportion of patients (2.8%) received low molecular weight heparins. Inferior vena cava filters were inserted in three (0.8%) patients.

**Fig. 1:**
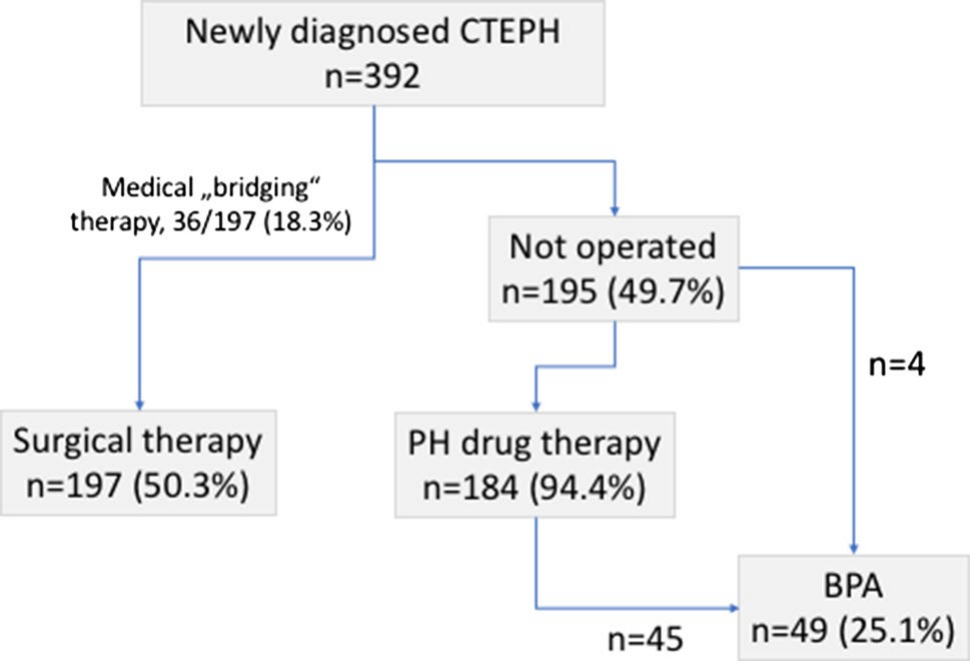
Treatment patterns in 392 patients with newly diagnosed chronic thromboembolic pulmonary hypertension (CTEPH). *PH* pulmonary hypertension, *BPA* balloon pulmonary angioplasty

Treatment pathways are shown in Fig. 1. A total of 197 (50.3%) patients underwent PEA surgery; 148 (75.1%) in Bad Nauheim, 30 (15.2%) in Homburg and 19 (9.6%) in Hannover. The overall perioperative mortality rate was 5/197 (2.5%). The perioperative mortality rates for the individual centers were 4/148 (2.7%) in Bad Nauheim, 0 (0%) in Homburg and 1/19 (5.3%) in Hannover.

PH targeted therapies were used in 36/197 (18.3%) patients who were later operated and in 184/195 (94.4%) of the non-operated patients. Reasons for withholding drug therapy in non-operated patients were primary BPA (*n* = 4), malignancy (*n* = 1), advanced left heart disease (*n* = 1), or were unknown (*n* = 5). Riociguat was the drug used predominantly as initial treatment (81.1% of the patients who received medical therapy), followed by phosphodiesterase 5 (PDE5) inhibitors (15.5%) and endothelin receptor antagonists (ERA; 3.4%).

Forty-nine patients underwent balloon pulmonary angioplasty (BPA). This represented 12.5% of the entire patient population and 25.1 of the non-operated patients. There were no deaths associated with BPA in these patients.

## Discussion

According to our data, 392 patients were newly diagnosed with CTEPH in 2016, resulting in a CTEPH incidence of 5.7 per million adults in Germany. This figure is considerably larger than the previously reported estimates of 1.75 per million and 0.9 per million from the United Kingdom and Spain, respectively [20, 21]. On the other hand, our numbers closely mirror those observed in France where approximately 300 patients are newly diagnosed with CTEPH each year [24], resulting in a rough estimate of the CTEPH incidence in France of 5–6 per million adults.

Although higher than previously reported, these figures are lower than one would expect if 3% of survivor of acute pulmonary embolism develop CTEPH as it has been suggested recently by Ende-Verhaar and co-workers [19]. Approximately, 56,000 patients are admitted each year to German hospitals for treatment of acute pulmonary embolism (http://www.destatis.de, assessed 8 November 2017), and approximately 80% of these patients (i.e., about 45,000 patients) are expected to be alive after 1 year [25]. If 3% of these patients were to develop CTEPH, we should have identified at least 1400 patients with newly diagnosed CTEPH. In fact, the numbers should have been even higher (i.e., approximately 1750 patients) as 25% of the CTEPH patients in our series had no history of venous thromboembolism. It is likely that these assumptions overestimate the incidence of CTEPH. Given the similar numbers of patients newly diagnosed with CTEPH in Germany and France, two countries with a well-developed health care system, it is possible that the risk of developing CTEPH after acute pulmonary embolism may be lower than previously reported, presumably at about 1%, although underestimation due to missing cases cannot be ruled out (see below).

In terms of age, sex, predisposing factors, baseline 6 min walking distance, functional class and haemodynamics, our patients were largely comparable to those reported previously from a European CTEPH registry [26]. The same was true for the proportion of patients with previous venous thromboembolism, which was approximately 75% in both series [26]. Still, some differences were observed. In the European CTEPH registry, vena cava filters were used in 12.4% of the patients [26], whereas only 0.3% of the patients in the present series received a vena cava filter. This may reflect regional differences but perhaps also a global decline in the use of vena cava filters for both acute pulmonary embolism [27] and CTEPH. Our data also suggest that direct oral anticoagulants are increasingly used in patients with CTEPH. To the best of our knowledge, this is the first large CTEPH series where these drugs were used more frequently than vitamin K antagonists. There is still a lack of data on the use of direct oral anticoagulants in patients with CTEPH. The same is basically true for the vitamin K antagonists, except that these compounds have been used for decades in this patient population.

In the above-mentioned European CTEPH registry [26], 56.8% of the patients underwent surgery, which was slightly higher than the 50.3% of the patients who underwent surgery in our series. However, some patients in our series were still undecided or under continued assessment for surgery when the database was closed. It is unlikely that the availability of BPA in Germany had a relevant impact on the numbers of surgical procedures as the participating centers offer BPA exclusively to non-operable patients [28]. The perioperative mortality rate after PEA of 2.5% was comparable to previous reports from other centers [5, 6].

Drug therapy targeting PH was used in less than 20% of patients who eventually underwent PEA surgery. This came as surprise as previous reports have been suggesting an increasing use of PH medications in patients with operable disease (20% in 2005 and 37% in 2007, respectively) [29]. We assume that the relatively infrequent use of PH drugs in operable patients in our series was due to the fact that the vast majority of patients had been referred to a CTEPH center for diagnostic work-up and that the time span between referral and admission was usually short, i.e., less than 4 weeks, in all three centers (data not shown). This, together with relatively short waiting times for surgery may have obviated the need for bridging therapies.

Our study has limitations, in particular that full datasets were not available from all patients and that we could include only patients who were referred to a CTEPH center or enrolled into COMPERA. We were unable to capture patients who were never referred to one of the participating centers. Thus, we cannot exclude the possibility that we have missed some cases and we cannot provide reasonable estimates on how many patients this might have been. Still, in countries like Germany with an advanced health care system and nationwide PH centres, there should not be many patients diagnosed with CTEPH who are not referred for further work-up and treatment. On the other hand, recent data from the US have shown that many patients with PH are not treated at specialized centres and that the diagnostic work-up of these patients is often incomplete [30]. This includes a low rate of ventilation–perfusion scanning, which remains the most sensitive tool to detect CTEPH [31]. Despite the lack of comparable data from Germany, it is likely that we face similar issues. Hence, our numbers reflect the most conservative estimate of the CTEPH incidence in Germany, and the true incidence may be even higher. Finally, we wish to emphasize that the reported treatments represent a snapshot of how the patients under study were managed in the centres. These data do not confer any recommendation regarding the best possible therapy for patients with newly diagnosed CTEPH.

In summary, the incidence of patients with an established CTEPH diagnosis in Germany was 5.7 per million adults in 2016. Although these numbers reflect a conservative estimate, they indicate that the incidence of CTEPH is higher than previously reported. Roughly half the patients underwent PEA surgery, whereas the remaining patients required targeted drug therapy and, occasionally, interventional treatment.
